# On the Time Course of Synchronization Patterns of Neuronal Discharges in the Human Brain during Cognitive Tasks

**DOI:** 10.1371/journal.pone.0063293

**Published:** 2013-05-16

**Authors:** Milan Brázdil, Jiří Janeček, Petr Klimeš, Radek Mareček, Robert Roman, Pavel Jurák, Jan Chládek, Pavel Daniel, Ivan Rektor, Josef Halámek, Filip Plešinger, Viktor Jirsa

**Affiliations:** 1 Behavioural and Social Neuroscience Research Group, CEITEC – Central European Institute of Technology, Masaryk University, Brno, Czech Republic; 2 Brno Epilepsy Center, Department of Neurology, St. Anne’s University Hospital and Medical Faculty of Masaryk University, Brno, Czech Republic; 3 Institute of Scientific Instruments, Academy of Sciences of the Czech Republic, Brno, Czech Republic; 4 Department of Physiology, Medical Faculty of Masaryk University, Brno, Czech Republic; 5 Institut de Neurosciences des Systèmes UMR1106 Inserm, Aix-Marseille Université, Marseille, France; University of British Columbia, Canada

## Abstract

Using intracerebral EEG recordings in a large cohort of human subjects, we investigate the time course of neural cross-talk during a simple cognitive task. Our results show that human brain dynamics undergo a characteristic sequence of synchronization patterns across different frequency bands following a visual oddball stimulus. In particular, an initial global reorganization in the delta and theta bands (2–8 Hz) is followed by gamma (20–95 Hz) and then beta band (12–20 Hz) synchrony.

## Introduction

During cognitive tasks, different brain regions communicate with each other via oscillatory signals with functionally differentiated frequency signatures [1], but the details of the mechanisms linking the cognitive dynamics to neural events are still unknown. Transient synchronization of neuronal discharges has been proposed as one possible mechanism to dynamically bind widely distributed sets of neurons into functionally coherent ensembles [2], [3]. In full compatibility, the communication-through-coherence hypothesis suggests that at the heart of cognitive dynamics lies a dynamic communication structure based on flexible neuronal coherence patterns [4]. Evidence for these hypotheses is found invasively in the cat and non-human primate brain [5]–[9] and non-invasively through EEG and MEG in the human brain [1], [3], [10]–[13]. In human studies, synchronization was consistently associated with an oscillatory patterning of neuronal responses, most often in the beta and gamma frequency range. Long-distance synchronization seemed to manifest itself in the lower frequency ranges such as beta, but also in the theta (4–8 Hz) and alpha (8–12 Hz) range [1]. Recently published studies on primates then proposed that frequency-specific neuronal correlations in large-scale cortical networks rather may be “fingerprints” of canonical neuronal computations underlying cognitive processes (for review see [14]). However, non-invasive scalp imaging in humans is a synthetic measure of multiple local circuits [12] and provides only limited information on the spatiotemporal evolution of the brain signals. For proper validation, invasive multisite studies are obligatory, but they are rather rare and mostly limited to a small number of subjects [13], [15]–[18]. Using various methodological approaches (e.g. computation of cross-correlations, coherence, phase synchrony, Granger causality, cross-frequency coupling, etc.) still some authors recently started to study changes in synchronization patterns in intracranial recordings in cognitive tasks [19]–[27]. As far as we are aware the most comprehensive cognitive study treating in a complex manner the brain dynamics in the cohort of ten invasively investigated human subjects is the study of Gaillard et al [28]. Here intracranial event-related potentials, event-related spectral perturbations, phase coherence and Granger causality were analyzed during visible and masked words processing. Partially in accordance with previous discoveries the authors found the local increases in spectral power in the gamma band and the significant increases in long-distance phase synchrony in the beta range during processing of consciously perceived words.

In the present study we investigate invasively, in a large cohort of human subjects, context-dependent global neural communications during a simple discrimination task with randomly presented rare and frequent visual stimuli. This cognitive paradigm known as a visual oddball task was previously extensively studied in both scalp and intracranial EEG recordings [29]–[36]. Research in several independent labs then clearly identified a set of cortical and subcortical generators of relevant event-related potentials (i.e. areas of the brain involved in the genesis of a set of cognitive processes during discrimination stimulus type), but functional integration among these sites was only rarely investigated within intracranial EEGs [18], [22], [37]–[39]. The data presented in this study are highly heterogeneous due to the significant inter-individual variability in number and locations of implanted electrode, hence we focus here on the temporal aspects of neural cross-talk and do not primarily consider the spatial aspects on the group level. This study offers a unique opportunity to identify and confirm some of the proposed principles on global information integration in the human brain.

## Methods

### Ethics Statement

Written informed consent was obtained from each subject prior to the investigation and the study received approval from the Ethics Committee of Masaryk University in Brno. Intracerebral EEG measures and their mathematical analyses were taken to document the process and the ethic committee approved this consent procedure. On the behalf of children participants involved in the study the written informed consent was provided by their caretakers.

### Subjects

Ten patients (six males and four females) ranging in age from 17 to 41 years (with an average age of 28.9 years, std. 8.21), all with medically intractable epilepsies, participated in the study (Table 1). Depth electrodes were implanted to localize seizure origin prior to surgical treatment. Each patient received 6–15 orthogonal platinum electrodes in the temporal and/or frontal, parietal, and occipital lobes using the stereotaxic coordinate system of Talairach [40]. Bilateral and multilobar investigations were conducted for most subjects. A total of 898 intracerebral sites were electrophysiologically investigated by means of 95 multicontact depth electrodes over the subjects (49 frontal, 31 temporal, 11 parietal, and 4 occipital). Standard semiflexible depth electrodes (ALCIS) with a diameter of 0.8 mm, a contact length of 2 mm, and an intercontact interval of 1.5 mm were used for invasive EEG monitoring. The exact positions of the electrode contacts in the brain were verified using postplacement MRI with electrodes in situ. Lesional anatomical structures and epileptogenic zone structures were not included in the analysis (recording sites from these structures are not included in Table 1 - last column). All subjects were on chronic anticonvulsant medication (usually slightly reduced due to the video-EEG monitoring) and all of them had normal or corrected-to-normal vision. All the patients were able to fully understand and perform the experimental task.

**Table 1:** Patient characteristics.

Subject no.	Sex	Age(years)	Dominanthand	MRI^a^	Epileptic focus^a^	Implantedsites^a^	No. ofrecording sites	No. of analyzedcontact pairs
1	M	36	R	normal	pericentral L	LFP, RFP	99	4,851
2	F	24	R	normal	precentral R	RFTP	105	5,460
3	M	41	R	HS LT	hippocampus L	LFTP, RFTP	92	4,186
4	M	23	R	FCD RF	frontopolar R	LFPTO, RFTP	108	5,778
5	M	29	R	normal	occipital L	LTPO, RTP	114	6,441
6	M	17	R	FCD L precentral	premotor L	LFP	58	1,653
7	F	41	R	normal	anterotemporal L	LFTP	87	3,741
8	F	29	R	DNET RT	mesiotemporal R	LT, RT	75	2,775
9	M	21	L	normal	insula R	LF, RFTP	109	5,886
10	F	28	R	EMC LT	mesiotemporal L	LT	51	1,275

a T, temporal; F, frontal; P, parietal; O, occipital; R, right; L, left; HS, hippocampal sclerosis; FCD, focal cortical displasia; DNET, dysembryoplastic neuroepitelial tumour; EMC, encephalomeningocele.

### Visual Oddball Task

Subjects were seated comfortably in a moderately lit room with a monitor screen positioned approximately 100 cm in front of their eyes. During the examination, they were requested to continuously focus their eyes on the small fixation point in the centre of the screen and to minimize blinking. A standard visual oddball task was performed: two types of stimuli (frequent and rare) were presented in the centre of the screen in random order. Clearly visible yellow capital letters O (frequent) and X (rare; approx. 50 trials) on a black background were used as experimental stimuli. The duration of stimuli exposure was constant at 500 ms; the ratio of rare to frequent stimuli was 1∶5. The interstimulus interval randomly varied between 4 and 6 s. Each subject was instructed to respond to the rare (target) stimulus as quickly and accurately as possible by pressing a microswitch button in the dominant hand.

### EEG Recordings

The EEG signal was simultaneously recorded from various intracerebral structures and a limited number of midline scalp electrodes (Fz, Cz, and Pz), using the 128 channel TrueScan EEG system (Deymed Diagnostic). All recordings were monopolar, with a linked earlobe reference. All impedances were less than 5 kΩ. Eye movements were recorded from a cathode placed 1 cm laterally and 1 cm above the canthus of the left eye, and from an anode 1 cm laterally and 1 cm below the canthus of the right eye. The sampling rate was 1024 Hz. Standard anti-aliasing filters were used. Occasional eye movements and muscle artefacts were off-line rejected manually and further processing was performed with artefact-free intracerebral EEG periods.

### Signal Processing

Data processing was carried out on monopolar montages with common reference and bipolar montages in order to distinguish between far field and local field effect to signal propagation. Bipolar montages were calculated by subtracting signal recorded from adjacent contacts belonging to the same intracerebral electrode. All the following processing is the same for both monopolar and bipolar montages.

Artefact-free intracerebral EEG trials with target and frequent stimuli were analyzed separately. The number of artefact-free frequent stimuli was randomly reduced to obtain the same number of trials for target and frequent stimuli for each subject. The number of analyzed trials varied from 30 to 48 depending on the subject. The EEG signal was passband filtered and analyzed in six frequency bands: δ (2–4 Hz), θ (4–8 Hz), α (8–12 Hz), β (12–20 Hz), lower γ (20–45 Hz), and upper γ (55–95 Hz). EEG signal was filtered before segmentation. Filter based on Fourier transformation was used.

### Cross-correlation Computation

The time cross-correlation of contact pairs, given by Pearson’s correlation coefficient, was computed in overlapping time window moving over the whole length of trials. The length of the time windows for correlation computing were 500, 250, 125, 80, 60, and 30 ms in frequency bands δ, θ, α, β, lower γ, and upper γ respectively. The length of the window was at least one period of the lowest frequency in the analyzed band. The shift of the moving window was tenth the window width. No mutual time shift was supposed between contacts in analyzed pairs. In this case the changes of correlation may have the origin in the change of signal shape or in time shift between channels. Within subjects, the number of cross-correlation contact pairs varied from 1,275 to 6,441.

### Power Envelope Computation

Signal’s power envelope within each trial was evaluated by Hilbert transform demodulation in selected frequency band. The stimuli-locked signals were particularly eliminated by subtracting averaged trial from all trials before demodulation computation.

### Statistics

The post-stimulus changes of correlation and power corresponding to the resting phase before the stimulation were evaluated. The statistically significant changes between the mean values in the reference period 100–700 ms before stimulus and the corresponding mean values in the moving window (one-third the width of the reference period) in the time area after stimuli were determined. The non-parametric Wilcoxon test for paired samples over trials was used. Statistical significance was computed separately for each subject and each frequency band, and for target and frequent stimuli. We adjusted p value to the number of contacts in each subject by the multiple-comparison Bonferroni correction. The post-stimulus statistical significance is the basis for the following analysis.

All processing was performed using ScopeWin and Matlab software.

### Graphical Interpretation of Cross-correlation

The results of the cross-correlation analysis were given in matrices in which lines correspond to contact pairs and columns correspond to time. Matrix values have only three levels representing statistically significant increase (red color), decrease (blue color) and no significant change of correlation (transparent) relative to baseline. The level of statistical significance was set to p<0.05 after Bonferroni correction.

**Figure 1:**
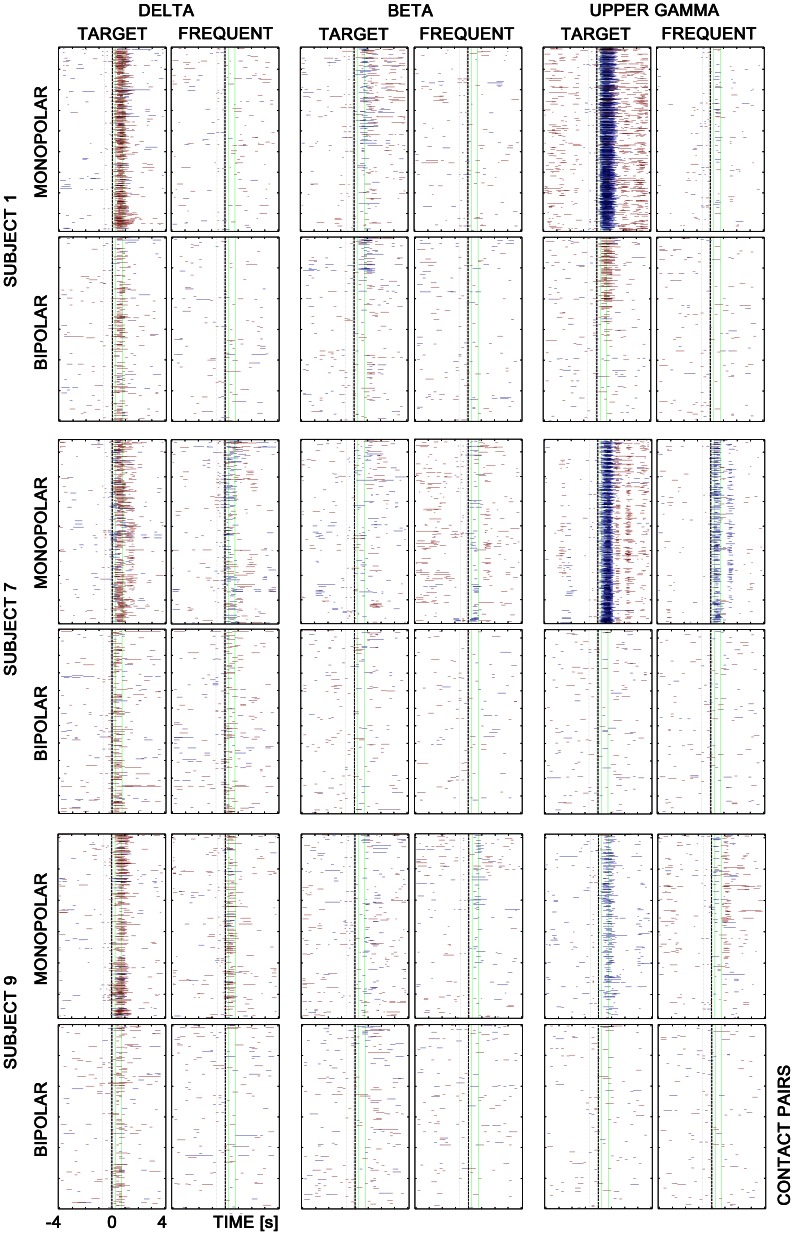
Matrices with significant correlation changes in three subjects; three frequency bands – δ (2–4 Hz), β (12–20 Hz), and upper γ (55–95 Hz), and two different stimuli – targets vs.frequents, for monopolar and bipolar montages. The correlation increase is highlighted in red, decrease in blue. Each colored line corresponds to changes in a contact pair in time (notice all pairs of investigated subject’s contacts are represented in individual matrices for each patient). Green vertical lines define interval 250–750 ms after stimuli.

Such matrices were computed separately for each subject and each frequency band, and for target and frequent stimuli. They provide a time overview in what latency relative to stimuli (zero in time axis) the correlation increase/decrease is significant. These matrices are used as the input data for graphic presentation (Fig. 1) and numerical analysis. Such a way of correlation representation offer clear information about time distribution. Unfortunately, the spatial localization is difficult to reach.

**Figure 2:**
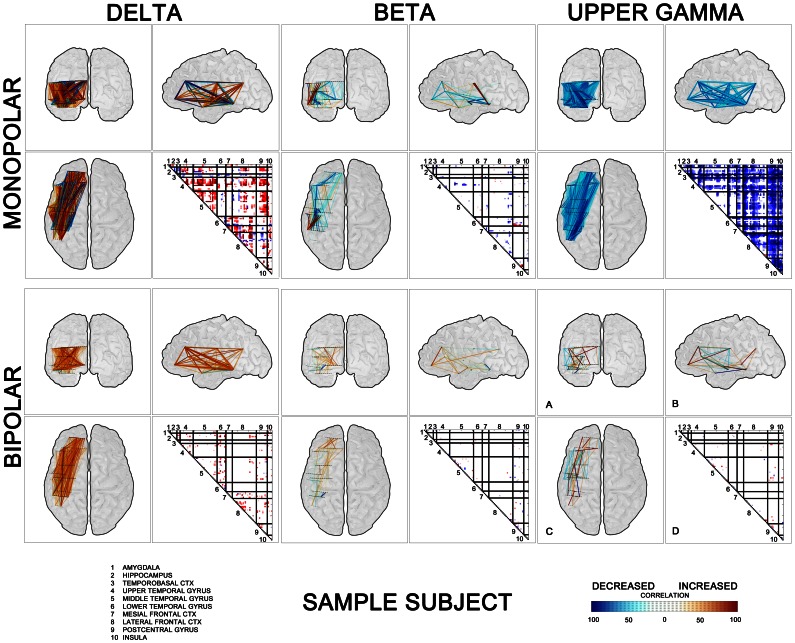
An example of spatial representation of post-stimulus interactions after targets between all investigated brain sites in one subject (No.7). Correlation results are arranged in the triangular matrices into groups according to brain structures (delimited by black lines)(D) and in graphic form of “glass brains” with linked pairs of investigated electrode contacts (A – Coronal, B – Sagittal, C – Axial). Matrix values and links are colored according to the percentage of duration of the increase (red) or decrease (blue) in cross-correlations within time window 250–750 ms after stimulation. Three selected frequency bands – δ (left panel), β (middle panel), and upper γ (right panel).

To treat a spatial interpretation of our results it is necessary to select the time interval, here 250–750 ms after stimuli was chosen. In this time window the effect of targets is expected based on previous intracranial event-related potentials, event-related synchronization/desynchronization and coupling studies [13], [17], [28], [41]. Then two approaches for graphical representation of results have been used (Fig. 2). First post-stimulus interactions in the chosen time interval after stimuli among all investigated brain sites were arranged into the individual triangular matrices (Fig. 2 D). The single value of triangular matrices was given by ratio of the length of time period that represents significant increase/decrease of correlation to the entire interval length (500 ms). Each contact pair lines (Fig. 1) were then represented by one point in triangular matrix. The relative value of increase/decrease of significant correlation changes over given time window was represented by the level of red/blue color. When the increase/decrease of correlation appeared in whole selected time window, the red/blue color was full dark. Any shorter increase/decrease was presented by lighter color, dependent on total percentage of significant points in the time window. White color expresses that no significant change of correlation occurs in given time window.

The second approach was to depict three orthogonal views of a transparent “glass brain" with significant cross-correlation changes [28] (Fig. 2 A,B,C). It is the same result as in triangular matrix including blue and red color meaning, but this approach might be better used for illustrating the results of spatial group analysis. All contact pairs spatially localized with significant decrease/increase of correlation are in glass brain linked with color lines.

**Figure 3:**
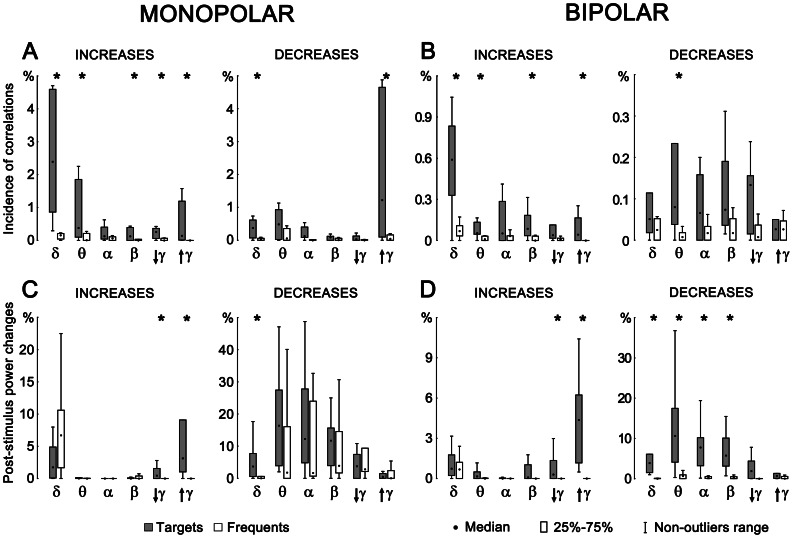
Relative incidences of significant post-stimulus changes in inter-areal cross-correlations (A – monopolar; B – bipolar) and post-stimulus power changes (C – monopolar; D – bipolar) across all investigated subjects. Statistical significance of target/frequent differences is indicated by asterisks.

Grand average across all subjects of cross-correlation and power changes according to stimulus (target vs. frequent) were computed (Fig. 3). Within long interval 0 to 1.5 s after stimuli and over all correlation pairs or single contact power the relative increase/decrease was calculated. This value, obtained for each subject separately, is given by dividing the area that represents significant increase/decrease by the entire area. Entire area is defined by interval length (1500 ms) and number of correlation pairs (Fig. 1) or contacts (power). A non-parametric test for paired samples was finally used to test statistical significance of relative cross-correlation and power changes between target and frequent stimulus over all subjects. Grand average was computed in six frequency bands δ, θ, α, β, lower γ, and upper γ.

## Results

Satisfactory behavioral performance of all subjects was observed during the experiment. The mean reaction time in the group of patients was 487 ms (std. 71.4). The mean accuracy of subjects’ responses was 99.5% (std. 1.1).

Significant changes (p<0.05) of inter-areal cross-correlations were detected after the stimuli in a number of investigated pairs for both monopolar and bipolar montages (Fig. 1). In several frequency ranges, the cross-correlations increased or decreased after targets significantly more often than after frequents. As expected these differences were more prominent in monopolar analyses, but they could be observed in bipolar montages as well. Figure 2 is giving a single subject example of spatial coupling after targets among different brain sites. The post-target interactions among investigated sites are both arranged here into the triangular matrices and depicted in three orthogonal views of a “glass brain”.

Critical across subject analysis revealed significant differences in oscillatory coupling after targets versus frequents quite congruently for both types of montages. The relative incidence of correlation increases was significantly higher for targets in δ, θ, β, and upper γ frequency bands with the most prominent findings in the δ range (Fig. 3A,B). In parallel, in some frequency ranges – δ and upper γ (for monopolar montages) and θ (for bipolar montages) – targets significantly more often than frequents decreased the inter-areal cross-correlations in other contact pairs. Importantly, the most frequent decreases were observed in the upper γ passband in monopolar signals, which however dramatically dropped after re-referencing.

The comparison of statistically significant target vs. frequent cross-correlations and power changes revealed certain relationships between the power increase (synchronization)/decrease (desynchronization) and change of both shape and time shift of signals represented here by cross-correlation (Fig. 3). The increases of power accompanied by the increase of correlation were only found in γ range. The decrease of power was dominant in δ, θ, α, and β bands, where parallel significant increase in correlation incidence was revealed in bipolar montages (Fig. 3 B,D). It can therefore be postulated that desynchronization here is associated with an increase in correlation.

**Figure 4:**
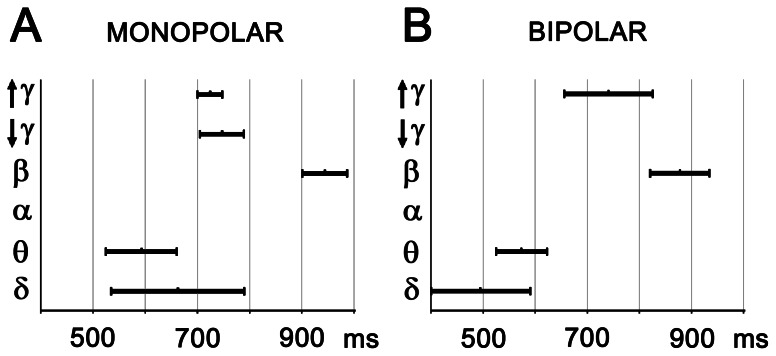
Timing of significant post-target cross-correlation increases across all subjects and areas. The duration was determined as the mean level of corresponding changes over contact pairs, and the occurrence was given as the center of changes.

The temporal characteristics of global cross-correlation changes after targets were as follows: the very first significant and massive change in terms of cross-correlation increase (reflecting inter-areal coupling) occurred in the delta and theta frequencies at about 500 ms after target and sustained for some 100–200 ms. Subsequently a significant coupling in gamma oscillations emerged at about 700 ms, followed by the final increase in global cross-correlation within the beta activities closed the event at about 1 second after target presentation (Fig. 4, Table 2). A non-parametric test for paired samples across all time appearing and patients showed that difference in the timing of cross-correlation increases in the beta frequency band was statistically significant at p<0.05 with Bonferroni correction.

**Table 2:** Temporal characteristics of significant cross-correlation changes after targets for monopolar (A) and bipolar montages (B).

A					
INCREASE	δ	θ	β	Lower γ	Upper γ
**Centre [ms]**	662 (135.8)	592 (188.8)	944 (97.0)	747 (171.5)	724 (103.6)
**Duration** **[ms]**	255 (81.2)	136 (25.0)	86 (29.7)	85 (29.9)	48 (49.1)
**DECREASE**	**δ**	**Upper γ**			
**Centre [ms]**	603 (200.0)	623 (57.3)			
**Duration** **[ms]**	195 (67.4)	100 (78.0)			
**B**					
**INCREASE**	**δ**	**θ**	**β**	**Upper γ**	
**Centre [ms]**	495 (100.4)	574 (289.6)	878 (386.0)	741 (235.6)	
**Duration** **[ms]**	193 (196.2)	98 (78.1)	115 (79.3)	170 (133.6)	
**DECREASE**	**θ**				
**Centre [ms]**	711 (223.5)				
**Duration** **[ms]**	126 (54.2)				

Standard deviations are given in brackets.

**Figure 5:**
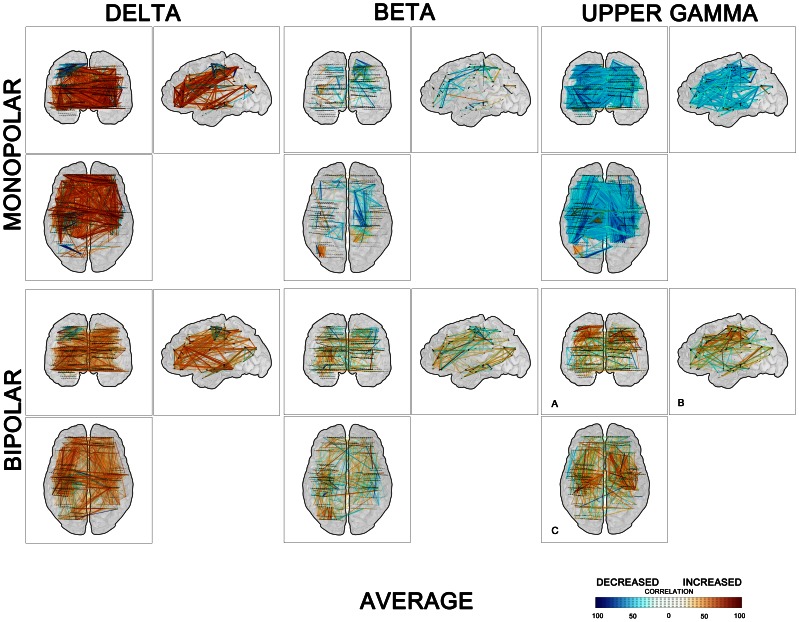
Spatial distribution of couplings/decouplings across all investigated subjects, in the time window 250–750 ms after targets. Three orthogonal views of a transparent “glass brain”; links are colored according to the percentage of duration of the increase (red) or decrease (blue) in cross-correlations within time window 250–750 ms after stimulation. Three selected frequency bands – δ (left panel), β (middle panel), and upper γ (right panel).

Figure 5 provide in a graphic form the values and the spatial distribution of the post-target cross-correlation changes across all subjects in the time window 250–750 ms after stimuli. This figure demonstrates a global pattern of both synchrony/desynchrony in all the depicted frequency ranges with maximum of long-distance (e.g. inter-hemispheric) couplings in the δ band. Further clearly dominant coupling over decoupling within the low frequencies can be observed whilst mid and upper frequencies reveal bigger amount of correlation decreases. And finally after the treatment of potential volume conduction (by bipolar montaging) a significant amount of long-distance mid and upper frequencies coupling/decoupling is still present.

## Discussion

In humans, large-scale neural network dynamics investigation is most frequently characterized by the neuroimaging data that can be acquired non-invasively such as electroencephalography, magnetoencephalography or functional magnetic resonance imaging. This macroscopic dynamics is by definition a synthetic measure of multiple local circuits and does not satisfactorily reveal the details of information processing in brain dynamics. It was therefore suggested that large-scale integration be examined optimally at the mesoscopic scale (among neural assemblies), which necessitates invasive recordings of EEG activity using intracerebral macro- or microelectrodes [12].

Our findings demonstrate in a large cohort of subjects, and at the optimal mesoscopic scale, the time course details of the synchronization patterns of neuronal discharges following a cognitively relevant stimulus. A broad spectrum of frequencies is involved with predominant coupling in slower frequencies and less expressed global synchrony in the middle and upper frequency passbands. Immediately after stimulus offset, during the initial phase of cognitive signal processing a highly significant reorganization of the couplings within the delta bands takes place. The inter-areal synchronization in this frequency band was present in the majority of investigated pairs with significant post-stimulus cross-correlation changes, and it was significantly more often after target than frequent stimuli. Much less frequently, in other investigated pairs or subjects, opposite inter-areal decoupling emerged in the same passband, which however did not significantly differ for targets and frequents. Given the hypothesis that slower oscillations are involved in long-range synchrony and the coordination of faster oscillations in functionally related but spatially segregated areas [8], [16], this finding support an initial reorganization of the network dynamics on a large scale across various brain areas. The significant increase in correlation within slow frequencies (including delta and theta bands) was previously observed between two distant brain areas in animal experiments during perception of stimuli with varying behavioral significance [8]. Also Canolty et al [22], focusing on changes in theta phase coupling in linguistic target detection task, observed strong target-selectivity within the widespread network of electrodes in one invasively investigated epileptic subject. What is however the exact timing of this long-distance delta/theta coupling, and what are the temporal relationships to the synchronization in other frequencies, was not treated in previous studies (to the best of our knowledge). Following this initial large-scale organization, a presumably more local reorganization of the couplings in the gamma bands take place, which will be related to more functionally distinct processes. The well-known local gamma synchronization, which is reflected in large post-target power increase, is however accompanied by more long-distance gamma synchrony in our study. Importantly, large-scale synchronies in the gamma band also have been observed within intracortical recordings from a single epileptic patient performing a visual discrimination task (almost identical to the ours) in the earlier methodological study of [18]. Theoretically the locally synchronized oscillatory responses can become synchronized over large distances due to reciprocal coupling of the oscillatory networks via excitatory cortico-cortical connections [42]. Then both local and long-range gamma couplings can together mediate bottom-up effects of behaviorally significant stimuli and may act as distributed unifying mechanism [8], [43], [44]. The final stage of cognitive processing reflected in EEG synchronization is characterized by the release of the couplings across beta band, which seems from previous primate studies to exert a final top-down modulation [45], [46]. In the extensive study of Gaillard et al., significant long-distance phase synchrony in the beta range was observed after presented words which increase was as the only one significantly correlated with conscious access to the stimuli [28]. In this study, which showed in a parallel way a significant increases of spectral power in the gamma band during conscious processing of the visible words, unfortunately slower frequencies (theta and delta) were not analyzed. Also no temporal code of various frequency couplings was addressed here.

The discussed form of time scale hierarchy is a well-known mechanism in dynamic system theory separating processes and functions within one and the same system. On the other hand our data does not correspond fully with earlier views on the inter-areal oscillatory frequency as a function of the distance only and rather shows that slower and faster oscillations might be involved in the same-range synchrony over the same Euclidean distances (Figs. 2 and 5). Even if synchronization among remote groups of neurons or among large assemblies of neurons truly tends to occur at lower oscillation frequencies than synchronization of local clusters of cells [42], still there exist, very likely functionally significant, gamma oscillations binding between remote areas too. This finding fits also well with repeatedly proven association of the cognitive process with long-range coherence in the gamma range [18], [24], [47]. The present study limitations are related to the a) recruitment of chronic epileptic patients (some of them with structural brain pathology) and all of them on chronic anticonvulsant medication, which makes the results difficult to generalize on the normal population, and b) to the available electrodes and related limited analysis between electrode contacts within a given patient which sometimes tend to be regrouped within a distinct cortical area and make it difficult to analyze frequency couplings across the majority of distant brain areas.

The interesting question is what all kinds of cognitive processes are actually reflected in observed synchronization patterns of neuronal discharges. For the average response time is shorter than the effect latencies (>500 ms), it is unlikely that our findings reflect a set of pre-movement cognitive functions, including early attentional, mnestic and executive processes. Rather we can speculate that they might mirror some broader aspects of cognitive processing, including more complex attentional functions, performance monitoring, and perhaps also affective processing related to successful rare stimulus detection.

It is intriguing, in light of the search for the link between cognitive dynamics and neural events, to find evidence for characteristic time courses of multi-frequency synchronization patterns of neural discharges in the human brain. This highly characteristic temporal structure of the evolution of couplings suggests that different frequency bands carry different dimensions of the integration process rather than only reflecting, as previously suggested, their dependence on the distance of structures that are involved [43], [48]. It is also congruent with the recently proposed view on the frequency-specific neuronal correlations in large-scale cortical networks that point to the underlying computations [14]. This “spectral fingerprint” very likely reflects all the complexity of the brain dynamics, including distinct biophysical properties of involved circuit mechanisms and many simultaneously engaged aspects of cognitive processes.

When interpreting the results, it is necessary to consider the differences between monopolar and bipolar montages in intracerebral EEG. Monopolar recordings represent the voltage referred to common reference in its absolute value. Resulting signal shape includes contribution of both local and far field sources. Very often power of far fields is much higher than local fields. Far field spatial distribution significantly increases correlation between remote areas as well as correlation after stimuli. Against, bipolar montages represent only differences between two closely adjoining contacts and not the actual value of ground referenced potential. These differences are often low voltage level contaminated by noise. It also depends on the polarity of the signal in bipolar subtraction. This bipolar montage features reduce the correlation value and changes correlation character. Bipolar correlation can be interpreted as coupling of “neighborliness”.

In the presented results we can find strong reduction of coupling occurrence in bipolar montages. Remarkable is the correlation diference in monopolar/bipolar montages in low and middle frequencies and upper gamma range. In monopolar upper gamma most often reveals the correlation decrease after target stimuli, but in bipolar there is in the upper gamma a significant correlation increase after targets (Fig. 3 A,B). Such diference we cannot find in other lower frequencies. Taking into account monopolar and bipolar properties we can speculate about strong local process activation (decrease of monopolar long-range correlation and increase of bipolar short-range correlation) after target stimulation in upper gamma range (Fig. 5, upper gamma). This finding confirms the hypothesis that faster oscillations are functionally related to spatially limited short-distance areas.

The important role in reactions to the cognitive stimulation seems to display simultaneous changes in correlation and power levels as well - monopolar/bipolar correlation/power relationship. The significant differences between the reactions to the targets and the reactions to the frequent stimuli indicate specific frequency bands (high for power increase - sychronization, low for power decrease - desynchronization), which are most likely taking place during decision-making process. In monopolar representation, revealed relationship between the synchronization/desynchronization and the phase coupling represented by correlation is showing that the increase of power in the delta band after target and frequent stimulation is supported by the increase of correlation only after target stimulation (Fig. 3 A,C, monopolar). The massive decrease of global cross-correlation after targets in the upper gamma appears to be connected to its significant power increase after target stimuli. Theta, alpha, and beta frequencies include predominant power decrease after both target and frequent stimuli. In case of bipolar montages however we can find predominantly significant increase in correlation incidence accompanied by decrease in desynchronization (Fig. 3 B increase, D decrease). The term significant here means significant increase of correlation after target stimuli in comparison with frequent and significant decrease of signal power after target stimuli in comparison with frequent over all subjects. This behavior is dominant for lower and mid frequencies - δ, θ, α, and β). We can assume that in the case of bipolar montages the increase in connectivity is associated with decrease of power - desynchronization. This feature appears only in bipolar montages and mainly represents connectivity to shorter distances. It also corresponds with Canolty et al. [22] results where theta power and phase coupling can change independently (dissociate).

These reactions might indicate the basic principles of mental activity in human brain. Patterns of synchronization and desynchronization evolve dynamically within the framework of a large-scale brain network. The essential ingredients determining the evolution of these oscillatory patterns are the connectivity and the time delays [49], [50], which are referred to as the space-time structure of the couplings of a network and are fundamental for synchronization/desynchronization.

Our present results are summarized over different structures within the human brain. The results show the overall feature of the brain activity including heterogeneous active areas. Essential information might be hidden in time as well and our results (rounded over whole time interval after the stimulation (from 0 to 1.5 s) could unfortunately provide us only with limited notion about correlation and power development over time only. The future depth EEG studies should focus on the synchronization patterns across different frequency bands within specific anatomical networks, should treat generally the interactions between different frequency bands and specifically between local and long-range gamma for instance, should examine shorter time intervals (and longer period) after the cognitive stimuli, and differentiate the impact of different cognitive tasks on the frequency-specific inter-areal correlations.

## References

[1] Schnitzler A, Gross J (2005) Normal and pathological oscillatory communication in the brain. Nat Rev Neurosci 6: 285–296. 15803160 10.1038/nrn1650

[2] Gray CM, Konig P, Engel AK, Singer W (1989) Oscillatory responses in cat visual-cortex exhibit inter-columnar synchronization which reflects global stimulus properties. Nature 338: 334–337. 2922061 10.1038/338334a0

[3] Singer W (1999) Neurobiology. Striving for coherence. Nature 397: 391–391. 29667958 10.1038/17021

[4] Fries P (2005) A mechanism for cognitive dynamics: neuronal communication through neuronal coherence. Trends Cogn Sci 9: 474–480. 16150631 10.1016/j.tics.2005.08.011

[5] Bressler SL (1996) Interareal synchronization in the visual cortex. Behav Brain Res 76: 37–49. 8734042 10.1016/0166-4328(95)00187-5

[6] Engel AK, Konig P, Kreiter AK, Singer W (1991) Interhemispheric synchronization of oscillatory neuronal responses in cat visual cortex. Science 252: 1177–1179. 2031188 10.1126/science.252.5009.1177

[7] Roelfsema PR, Engel AK, Konig P, Singer W (1997) Visuomotor integration is associated with zero time-lag synchronization among cortical areas. Nature 385: 157–161. 8990118 10.1038/385157a0

[8] von Stein A, Chiang C, Konig P (2000) Top-down processing mediated by interareal synchronization. Proc Natl Acad Sci U S A 97: 14748–14753. 11121074 10.1073/pnas.97.26.14748 PMC18990

[9] Canolty RT, Ganguly K, Kennerley SW, Cadieu CF, Koepsell K, et al (2010) Oscillatory phase coupling coordinates anatomically dispersed functional cell assemblies. Proceedings of the National Academy of Sciences of the United States of America 107: 17356–17361. 20855620 10.1073/pnas.1008306107 PMC2951408

[10] Sarnthein J, Petsche H, Rappelsberger P, Shaw GL, von Stein A (1998) Synchronization between prefrontal and posterior association cortex during human working memory. Proc Natl Acad Sci U S A 95: 7092–7096. 9618544 10.1073/pnas.95.12.7092 PMC22750

[11] Sauseng P, Klimesch W (2008) What does phase information of oscillatory brain activity tell us about cognitive processes? Neurosci Biobehav Rev 32: 1001–1013. 18499256 10.1016/j.neubiorev.2008.03.014

[12] Varela F, Lachaux JP, Rodriguez E, Martinerie J (2001) The brainweb: phase synchronization and large-scale integration. Nat Rev Neurosci 2: 229–239. 11283746 10.1038/35067550

[13] von Stein A, Rappelsberger P, Sarnthein J, Petsche H (1999) Synchronization between temporal and parietal cortex during multimodal object processing in man. Cereb Cortex 9: 137–150. 10220226 10.1093/cercor/9.2.137

[14] Siegel M, Donner TH, Engel AK (2012) Spectral fingerprints of large-scale neuronal interactions. Nature Reviews Neuroscience 13: 121–134. 22233726 10.1038/nrn3137

[15] Axmacher N, Schmitz DP, Wagner T, Elger CE, Fell J (2008) Interactions between medial temporal lobe, prefrontal cortex, and inferior temporal regions during visual working memory: a combined intracranial EEG and functional magnetic resonance imaging study. J Neurosci 28: 7304–7312. 18632934 10.1523/JNEUROSCI.1778-08.2008 PMC6670397

[16] Bruns A, Eckhorn R (2004) Task-related coupling from high- to low-frequency signals among visual cortical areas in human subdural recordings. Int J Psychophysiol 51: 97–9116. 14693360 10.1016/j.ijpsycho.2003.07.001

[17] Lachaux JP, Rodriguez E, Martinerie J, Adam C, Hasboun D, et al (2000) A quantitative study of gamma-band activity in human intracranial recordings triggered by visual stimuli. Eur J Neurosci 12: 2608–2622. 10947835 10.1046/j.1460-9568.2000.00163.x

[18] Lachaux JP, Rodriguez E, Martinerie J, Varela FJ (1999) Measuring phase synchrony in brain signals. Human Brain Mapping 8: 194–208. 10619414 10.1002/(SICI)1097-0193(1999)8:4<194::AID-HBM4>3.0.CO;2-C PMC6873296

[19] Dalal SS, Guggisberg AG, Edwards E, Sekihara K, Findlay AM, et al (2008) Five-dimensional neuroimaging: Localization of the time-frequency dynamics of cortical activity. Neuroimage 40: 1686–1700. 18356081 10.1016/j.neuroimage.2008.01.023 PMC2426929

[20] Canolty RT, Knight RT (2010) The functional role of cross-frequency coupling. Trends in Cognitive Sciences 14: 506–515. 20932795 10.1016/j.tics.2010.09.001 PMC3359652

[21] Axmacher N, Henseler MM, Jensen O, Weinreich I, Elger CE, et al (2010) Cross-frequency coupling supports multi-item working memory in the human hippocampus. Proceedings of the National Academy of Sciences of the United States of America 107: 3228–3233. 20133762 10.1073/pnas.0911531107 PMC2840289

[22] Canolty RT, Cadieu CF, Koepsell K, Ganguly K, Knight RT, et al (2012) Detecting event-related changes of multivariate phase coupling in dynamic brain networks. Journal of Neurophysiology 107: 2020–2031. 22236706 10.1152/jn.00610.2011 PMC3331660

[23] Womelsdorf T, Schoffelen JM, Oostenveld R, Singer W, Desimone R, et al (2007) Modulation of neuronal interactions through neuronal synchronization. Science 316: 1609–1612. 17569862 10.1126/science.1139597

[24] Gregoriou GG, Gotts SJ, Zhou HH, Desimone R (2009) High-Frequency, Long-Range Coupling Between Prefrontal and Visual Cortex During Attention. Science 324: 1207–1210. 19478185 10.1126/science.1171402 PMC2849291

[25] Korzeniewska A, Crainiceanu CM, Kus R, Franaszczuk PJ, Crone NE (2008) Dynamics of Event-Related Causality in brain electrical activity. Human Brain Mapping 29: 1170–1192. 17712784 10.1002/hbm.20458 PMC6870676

[26] Vidal JR, Freyermuth S, Jerbi K, Hamame CM, Ossandon T, et al (2012) Long-Distance Amplitude Correlations in the High Gamma Band Reveal Segregation and Integration within the Reading Network. Journal of Neuroscience 32: 6421–6434. 22573665 10.1523/JNEUROSCI.4363-11.2012 PMC6621125

[27] Canolty RT, Soltani M, Dalal SS, Edwards E, Dronkers NF, et al (2007) Spatiotemporal dynamics of word processing in the human brain. Front Neurosci 1: 185–196. 18982128 10.3389/neuro.01.1.1.014.2007 PMC2518055

[28] Gaillard R, Dehaene S, Adam C, Clemenceau S, Hasboun D, et al (2009) Converging Intracranial Markers of Conscious Access. Plos Biology 7: 472–492. 10.1371/journal.pbio.1000061 PMC2656551 19296722

[29] Halgren E, Squires NK, Wilson CL, Rohrbaugh JW, Babb TL, et al (1980) Endogenous potentials generated in the human hippocampal-formation and amygdala by infrequent events. Science 210: 803–805. 7434000 10.1126/science.7434000

[30] Wood CC, Allison T, Goff WR, Williamson PD, Spencer DD (1980) On the neural origin of P300 in man. Prog Brain Res 54: 51–56. 7220960 10.1016/S0079-6123(08)61605-2

[31] Wood CC, McCarthy GA (1985) A possible frontal lobe contribution to scalp P300. Society for Neurscience Abstracts 11: 879.

[32] Halgren E, Stapelton JM, Smith M, Altafullah I (1986) Generators of the human scalp P3(s) evoked potentials. Frontiers of Clinical Neurosciences 3: 269–284.

[33] McCarthy G, Wood CC (1987) Intracranial recordings of endogenous ERPs in humans. Electroencephalogr Clin Neurophysiol Suppl 39: 331–337. 3477444

[34] Brazdil M, Rektor I, Dufek M, Daniel P, Jurak P, et al (1999) The role of frontal and temporal lobes in visual discrimination task - depth ERP studies. Neurophysiologie Clinique-Clinical Neurophysiology 29: 339–350. 10546252 10.1016/s0987-7053(99)90047-3

[35] Polich J (2007) Updating p300: An integrative theory of P3a and P3b. Clinical Neurophysiology 118: 2128–2148. 17573239 10.1016/j.clinph.2007.04.019 PMC2715154

[36] Bekinschtein TA, Dehaene S, Rohaut B, Tadel FO, Cohen L, et al (2009) Neural signature of the conscious processing of auditory regularities. Proceedings of the National Academy of Sciences of the United States of America 106: 1672–1677. 19164526 10.1073/pnas.0809667106 PMC2635770

[37] Brazdil M, Babiloni C, Roman R, Daniel P, Bares M, et al (2009) Directional Functional Coupling of Cerebral Rhythms Between Anterior Cingulate and Dorsolateral Prefrontal Areas During Rare Stimuli: A Directed Transfer Function Analysis of Human Depth EEG Signal. Human Brain Mapping 30: 138–146. 17999400 10.1002/hbm.20491 PMC6870726

[38] Kukleta M, Brazdil M, Roman R, Bob P, Rektor I (2009) Cognitive Network Interactions and Beta 2 Coherence in Processing Non-Target Stimuli in Visual Oddball Task. Physiological Research 58: 139–148. 18198993 10.33549/physiolres.931404

[39] Kukleta M, Bob P, Brazdil M, Roman R, Rektor I (2009) Beta 2-Band Synchronization during a Visual Oddball Task. Physiological Research 58: 725–732. 19093717 10.33549/physiolres.931629

[40] Talairach J (1967) Atlas d’anatomie stéréotaxique du télencéphale: études anatomo-radiologiques: Masson.

[41] Halgren E, Marinkovic K, Chauvel P (1998) Generators of the late cognitive potentials in auditory and visual oddball tasks. Electroencephalography and Clinical Neurophysiology 106: 156–164. 9741777 10.1016/s0013-4694(97)00119-3

[42] Singer W (2009) Distributed processing and temporal codes in neuronal networks. Cogn Neurodyn 3: 189–196. 19562517 10.1007/s11571-009-9087-z PMC2727167

[43] von Stein A, Sarnthein J (2000) Different frequencies for different scales of cortical integration: from local gamma to long range alpha/theta synchronization. International Journal of Psychophysiology 38: 301–313. 11102669 10.1016/s0167-8760(00)00172-0

[44] Rodriguez E, George N, Lachaux JP, Martinerie J, Renault B, et al (1999) Perception’s shadow: long-distance synchronization of human brain activity. Nature 397: 430–433. 9989408 10.1038/17120

[45] Buschman TJ, Miller EK (2007) Top-down versus bottom-up control of attention in the prefrontal and posterior parietal cortices. Science 315: 1860–1862. 17395832 10.1126/science.1138071

[46] Saalmann YB, Pigarev IN, Vidyasagar TR (2007) Neural mechanisms of visual attention: How top-down feedback highlights relevant locations. Science 316: 1612–1615. 17569863 10.1126/science.1139140

[47] Siegel M, Donner TH, Oostenveld R, Fries P, Engel AK (2008) Neuronal Synchronization along the Dorsal Visual Pathway Reflects the Focus of Spatial Attention. Neuron 60: 709–719. 19038226 10.1016/j.neuron.2008.09.010

[48] Penttonen M, Buzsaki G (2003) Natural logarithmic relationship between brain oscillators. Thalamus & Related Systems 2: 145–152.

[49] Ghosh A, Rho Y, McIntosh AR, Kotter R, Jirsa VK (2008) Noise during Rest Enables the Exploration of the Brain’s Dynamic Repertoire. Plos Computational Biology 4. 10.1371/journal.pcbi.1000196 PMC2551736 18846206

[50] Deco G, Jirsa VK, McIntosh AR (2011) Emerging concepts for the dynamical organization of resting-state activity in the brain. Nature Reviews Neuroscience 12: 43–56. 21170073 10.1038/nrn2961

